# Structure-dependent antiviral activity of catechol derivatives in pyroligneous acid against the encephalomycarditis virus

**DOI:** 10.1039/c8ra07096b

**Published:** 2018-10-22

**Authors:** Ruibo Li, Ryo Narita, Ryota Ouda, Chihiro Kimura, Hiroshi Nishimura, Mitsuyoshi Yatagai, Takashi Fujita, Takashi Watanabe

**Affiliations:** Research Institute for Sustainable Humanosphere, Kyoto University Uji Kyoto 611-0011 Japan twatanab@rish.kyoto-u.ac.jp; Institute for Frontier Life and Medical Science, Kyoto University Kyoto 606-8507 Japan; Tokyo University Hongo, Bunkyo-ku Tokyo 113-8654 Japan

## Abstract

The pyrolysis product, wood vinegar (WV), from Japanese larch exhibited strong antiviral activity against the encephalomycarditis virus (EMCV). Catechol, 3-methyl-, 4-methyl-, 4-ethyl-, and 3-methoxycatechol, and 2-methyl-1,4-benzenediol were identified as the major antiviral compounds. The viral inhibition ability of these compounds was affected by the structure and position of the substituent group attached to the aromatic skeleton. The IC_50_ of catechol was 0.67 mg mL^−1^ and those of its derivatives were <0.40 mg mL^−1^. Methyl and ethyl substitution in the para position relative to a hydroxyl group obviously increased the antiviral activities. The mode of antiviral action was investigated by adding catechol derivatives at different times of the viral life cycle. It was found that direct inactivations of EMCV by these compounds were the major pathway for the antiviral activity. The effect of catechol derivatives on the host immune system was studied by quantification of Il6 and Ifnb1 expression levels. Increased Il6 expression levels indicate NF-κB activation by reactive oxygen species from auto-oxidations of catechol derivatives, which is also a possible antiviral route. The present research provides indices for production of potent antiviral agents form lignocellulose biomass.

## Abstract 2

The structure-related antiviral activities of catechol and derivatives were investigated. A new strategy for producing antiviral compounds was proposed.

## Graphical abstract

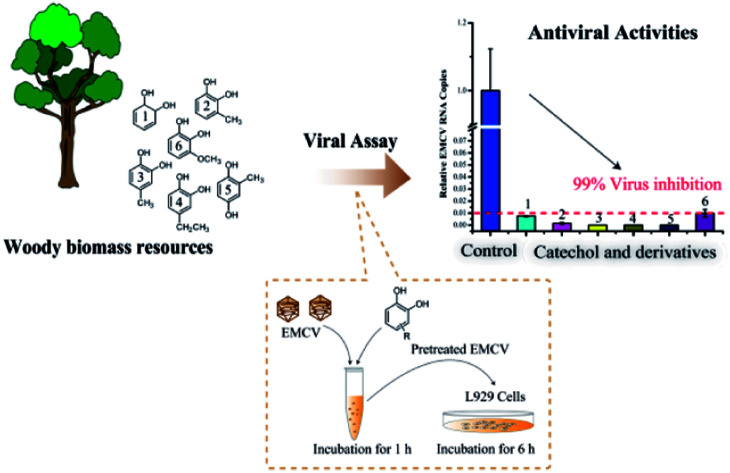

## Introduction

Recently, the demand for bioactive compounds derived from medicinal plants for pharmaceutical and cosmetic products as well as health food is increasing.^1^ Traditionally, bioactive compounds were obtained from medicinal plants by extraction and isolation, which is a time-consuming, low-yield, and high-cost process. In addition, the number of fields that can be used for natural plant growth and cultivation are decreasing year by year, and cultivation and growth of natural plants take several to tens of years. Thus, it is necessary to find other ways of obtaining bioactive compounds. Biomass, a naturally abundant and sustainable resource, includes a great number of products, such as agriculture, forest, and animal husbandry waste.^2^ More than 10 billion tons of biomass are produced globally each year.^3^ Utilization and conversion of biomass into chemicals has become an important research topic.^4–6^ Pyrolysis, a thermal biomass decomposition process that proceeds at intermediate temperature under low-oxygen conditions to yield bio-oil, gases, and biochar, is efficient for biomass conversion.^7,8^ The gas product, pyroligneous acid (PA), also called wood vinegar (WV), has been reported to show bioactivities such as anti-oxidant, anti-fungi, and anti-inflammation activity.^9–11^

As viruses threaten human health and cause huge economic losses, much effort has been focused on the discovery of antiviral drugs. Wood and bamboo vinegars have been reported to directly inactivate viruses, such as porcine reproductive and respiratory syndrome virus.^12^ Nevertheless, the antiviral compounds in wood and bamboo vinegar are still unknown. The encephalomyocarditis virus (EMCV), a small non-enveloped single-stranded RNA virus,^13,14^ belongs to the picornaviridae family. EMCV is very resilient, even in hostile conditions.^13^ For many mammalian species, infection with EMCV leads to several conditions including myocarditis, encephalitis, neurological diseases, reproductive disorders, and diabetes.^13^ However, antiviral strategies against EMCV are very limited.^15–17^ Our previous research demonstrated that bamboo vinegar from Moso bamboo showed strong antiviral activity against EMCV because of the synergistic effect of phenol with acetic acid.^18^ The composition and antiviral activity of WV depend on a number of factors that include the plant species and the pyrolysis conditions. The interactions between the constituent compounds and their respective biological activities are not yet well understood. In our recent research,^19^ we characterized and quantified the phenolic compounds in WVs derived from hardwood, softwood, and bamboo, and investigated their antiviral activities against EMCV. Their structure-activity relation was also elucidated in detail. However, the antiviral mechanism of these phenolic compounds is still not clear.

In this study, we fractionated WV derived from *Larix kaempferi* according to the antiviral activity. Catechol and its derivatives were identified to be the main viral inhibition compounds. To understand how catechol derivatives interact with EMCV, these compounds were added at different stages of viral infection. The effects of catechol derivatives on the host immune system were also investigated.

## Material and methods

### Wood vinegar and chemicals

WV derived from Japanese larch (*Larix kaempferi*) was obtained from Shimokawa Forest Association (Hokkaido, Japan), which was produced according to the guidelines of the Japan Mokusaku-eki Association.^18,19^ The concentration of acetic acid was 1.2% (weight percentage) and the pH was 3.22 at 20 °C. Catechol, 3-methylcatechol, 4-methylcatechol, and 1,2,4-trimethoxybenzene were obtained from Tokyo Chemical Industry Co., Ltd. (Tokyo, Japan). 2-Methoxy-4-methylphenol, 4-ethylcatechol, and 2-methyl-1,4-benzenediol were purchased from Wako Pure Chemical Industry Co., Ltd. (Osaka, Japan). 3-Methoxycatechol was obtained from Alfar Aesa (Tokyo, Japan). All the chemicals in this research had purities greater than 99.0%.

### Fractionation of wood vinegar

**Fig. 1:**
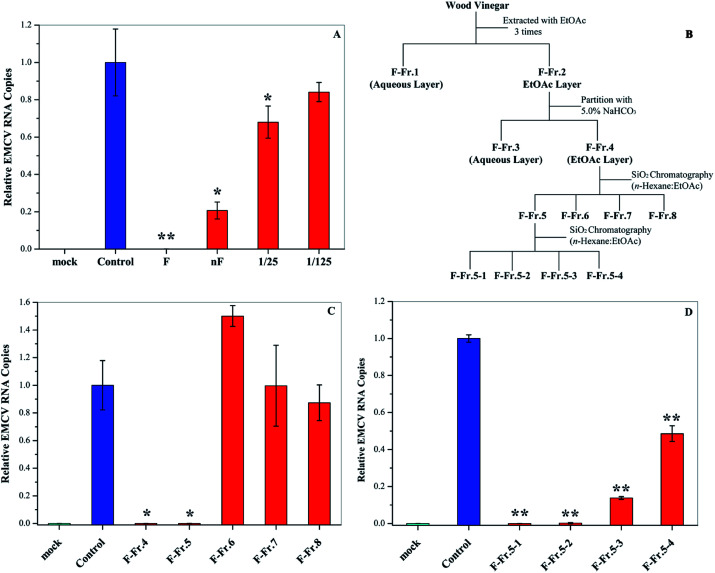
Antiviral activities of WV and its fractions. (A) Antiviral activities of original (F) and neutralized WVs (nF) as well as 25- and 125-fold dilution of nF. (B) Flowchart for the fractionation of WV F. Antiviral activities of (C) F-Fr.4 to F-Fr.8 at 2.0% and (D) F-Fr.5-1 to F-Fr.5-4 at 1.0%. *P < 0.05, **P < 0.01 indicate a significant difference compared to the solvent control by student's *t*-test.

Fractionation of WV was carried out according to previous research.^11,18^ WV F (1.0 L) was filtered with 5.0 and 0.45 μm membranes (Merck Millipore Ltd, Japan), respectively, and was then fractionated as shown in Fig. 1. First, liquid–liquid extraction was carried out using EtOAc (350 mL × 3 times) to generate an aqueous phase (F-Fr.1) and an organic phase (F-Fr.2). The organic phase (F-Fr.2) was then concentrated to 500 mL by evaporation. F-Fr.3 (aqueous phase) and F-Fr.4 (4.6814 g, organic phase) were obtained by partitioning F-Fr.2 with 5.0% NaHCO_3_. F-Fr.4 was fractionated by silica column (Biotage SNAP Ultra, Japan) chromatography using a mixture of *n*-hexane and EtOAc as the mobile-phase solvent to produce F-Fr.5 (2.4014 g), F-Fr.6 (0.7812 g), F-Fr.7 (169.4 mg), and F-Fr.8 (209.4 mg). One part of F-Fr.5 (1.8973 g) was separated by gradient in the same kind of silica gel column with the same mobile-phase solvent using the following procedure: 30 : 70 (2.0 CV (column volume)); the concentration gradient was increased from 30 : 70 to 70 : 30 in 5.0 CV; 70 : 30 (1.5 CV); 70 : 30 EtOAc : MeOH (2.0 CV) to generate F-Fr.5-1 (612.7 mg), F-Fr.5-2 (158.3 mg), F-Fr.5-3 (602.6 mg), and F-Fr.5-4 (44.3 mg).

### Gas chromatography-mass spectrometry

The original WV and fractions were analyzed by gas chromatography-mass spectrometry (GC-MS) according to previous report.^19^

The chemical compounds in the WV were identified by comparing their retention time and the mass spectra peaks with authentic standards. Catechol, 3-methylcatechol, 4-methylcatechol, and 4-ethylcatechol were quantified by GC-MS using butylated hydroxytoluene (BHT) as an internal standard. Calibration curves were determined using the peak area ratios of target compounds to the internal standard as the *Y*-axis and concentrations of the compounds as the *X*-axis. Six different concentrations (0.01, 0.1, 0.25, 0.75, 1.0, and 1.5 mg mL^−1^) were measured for the calibration curves. Correlation coefficients (*R*^2^) for all the curves were higher than 0.995.

### Cell culture

L929 cells (murine fibroblast cell line purchased from ATCC) were by using the same procedure reported previously.^19^

### Viral inactivation assay

EMCV was propagated in Vero cells, and its titer was determined by standard plaque assay on L929 cells as described previously.^20^ For the inactivation assay, 10 μL of medium (MEM) containing 1 × 10^5^ pfu EMCV was incubated with 10 μL of PA or catechol derivatives on ice for 1 h. Methanol (for catechol derivatives) or H_2_O (for wood PAs) was used as a solvent control. A total of 80 μL of culture medium was added to the mixture, and 10 μL of the mixture was added to 1 mL of culture medium containing 2.5 × 10^5^ L929 cells in a 12-well plate. After 6 h of incubation, the cells were harvested and subjected to real-time quantitative polymerase chain reaction (qPCR). The IC_50_ value of a compound was defined as the concentration at which it inactivated the relative EMCV RNA levels of the treated cells by 50%.

### Quantitative real-time qPCR

The total RNA of EMCV-infected cells was extracted with Sepazol (Nacalai tesque). A High-Capacity cDNA Reverse Transcription Kit (Applied Biosystems) was used for cDNA synthesis. Viral RNA levels were monitored with a StepOnePlus real-time PCR system (Applied Biosystems) and Fast SYBR Green Master Mix (Applied Biosystems) using the following EMCV-specific primers: forward 5′-TTA-TAG-TGC-CGG-ACC-TGG-CA-3′ and reverse 5′-CCC-AAG-CTC-CCA-GTG-TTG-TC-3′. The RNA copy number of EMCV was normalized to that of internal β-Actin: forward 5′-GAC-ATG-GAG-AAG-ATC-TGG-CAC-CAC-A-3′ and reverse 5′-ATC-TCC-TGC-TCG -AAG-TCT-AGA-GCA-A-3′.

### Amido black staining

The cells were washed in phosphate buffer saline (PBS, pH 7.4, Nacalai Tesque) and fixed with methanol. Then, 0.5% (w/v) Amido Black solution (Nacalai Tesque) was added to stain the cell. After 20 min of incubation at room temperature, the Amido Black solution was removed and the dye was eluted with 0.1 mol L^−1^ NaOH. Absorption was measured at 630 nm. The 50% cytotoxic concentration against the L929 cell (CC_50_) of a compound was defined as the concentration at which it reduced the absorbance at 630 nm by 50%.

### Quantification of cytokines (Ifnb1 and Il6)

L929 cells were seeded at 2.5 × 10^5^ in 12-well plates, cultured for 24 h, and then treated with catechol derivatives (25 μg mL^−1^) for 3 h at 37 °C. The cells were harvested and the total RNA was extracted with Sepazol (Nacalai tesque). A High-Capacity cDNA Reverse Transcription Kit (Applied Biosystems) was used for cDNA synthesis. Ifnb1 and Il6 RNA levels were monitored with a StepOnePlus real-time PCR system (Applied Biosystems) and Fast TaqMan Master Mix (Applied Biosystems) using the Ifnb1-and Il6-specific TaqMan probe and primers.

### Determination of ROS

L929 cells were seeded at 5 × 10^5^ in 6-well plates, cultured for 24 h, and then treated with catechol derivatives for 3 h at 37 °C. The medium was removed and cells were incubated with 1.0 mL of 5.0 μM MitoSOX™ (Invitrogen) HBSS/Ca/Mg for 10 min at 37 °C. The cells were washed three times with PBS. Cellular fluorescence images were analyzed using a microscope (Leica) with a 510 nm laser and 580 nm filter. Quantifications of ROS were performed using a FACS verse system (Becton Dickinson Biosciences) and FLOWJO software.

### Statistical analysis

The results were shown as mean ± SD of at least duplicate independent experiments. Student's *t*-test was used to determine statistical significance. *P* < 0.05 was considered significant.

## Results and discussion

### Anti-EMCV activities of wood vinegar

In this research, we examined the viral inactivation ability of WV derived from *Larix kaempferi* against EMCV. The results showed that the original WV (F) completely inactivated EMCV (Fig. 1A). It has been reported that organic acids, the main components in WVs,^21^ may affect the viral inhibition effect of WVs.^18^ In order to exclude the effect of organic acids, the original wood vinegar (F) was neutralized to pH 7.0 with NaHCO_3_ and the antiviral activity was assayed. After neutralization, the viral inactivation ability of WV decreased but still inhibited virus replication by about 80%. The neutralized WV (nF) inhibited EMCV in a dose–dependent manner (Fig. 1A). These results indicate that WV F contains some antiviral compounds.

### Fractionation of wood vinegar

The WV was fractionated as shown in Fig. 1B. First, liquid–liquid extraction was performed to generate aqueous F-Fr.1 and organic F-Fr.2 fractions, both of which can inactivate EMCV. The main components of F-Fr.1 were organic acids and some highly polar compounds. The organic phase F-Fr.2 was partitioned with a NaHCO_3_ solution to produce the acid fraction F-Fr.3 and the phenolic neutral fraction F-Fr.4. Further fractionation of F-Fr.4 resulted in F-Fr.5 to F-Fr.8. Strong viral inhibition effects were observed for F-Fr.5 (Fig. 1C), which was then separated to F-Fr.5-1 to F-Fr.5-4. F-Fr.5-1 and F-Fr.5-2 significantly decreased the ECMV infectivity (Fig. 1D). Therefore, it is evident that the antiviral components were highly concentrated in F-Fr.5-1 and F-Fr.5-2. GC-MS was used to identify and quantify the active antiviral compounds in these two fractions.

### GC-MS analysis of F-Fr.5-1 and F-Fr.5-2

**Fig. 2:**
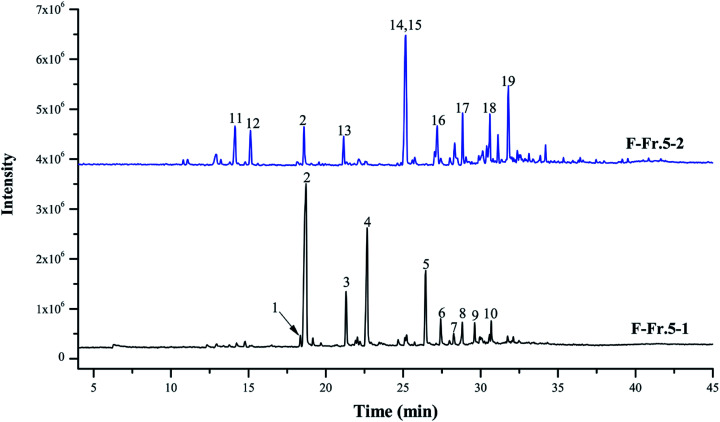
Total ion chromatograms of F-Fr.5-1 and F-Fr.5-2. The chemicals indicated by numbered peaks are listed in Tables 1 and 2.

The chemical compositions of F-Fr.5-1 and F-Fr.5-2 were analyzed by GC-MS (Fig. 2). The relative contents of the components were calculated by normalization of the peak area. Nineteen compounds were tentatively identified in F-Fr.5-1 and F-Fr.5-2, as shown in Fig. 2, Tables 1 and 2. Catechol, 3-methylcatechol, 4-methylcatechol, and 4-ethylcatechol were the main components in F-Fr.5-1 and their total amounts were higher than 60%. These results suggest that catechol and its derivatives are responsible for the antiviral activity of F-Fr.5-1. The concentrations of these compounds in F-Fr.5-1 and original WV F were quantified by GC-MS internal standard methods (Table 1). F-Fr.5-2 also markedly inactivated EMCV replication. Three kinds of catechol derivatives, catechol, 2-methyl-1,4-benzenediol, and 3-methoxycatechol, were identified (Table 2), and the concentrations were 0.20, 0.12, and 0.40 mg mL^−1^, respectively, in F-Fr.5-2. Woody biomass undergoing pyrolysis at high temperature forms different compounds. Cellulose and hemicelluloses are decomposed into ketones, alcohols, furan, and pyran derivatives, while lignin is mainly converted into phenol, guaiacol, syringol, and their derivatives.^22,23^ Catechol and its derivatives are not a component of natural lignin and are generated by secondary decomposition of lignin fragments such as guaiacol at high temperature (350–450 °C) and long times.^24,25^

**Table 1:** Catechol and catechol derivatives in F-Fr.5-1 and WV

No	Compounds	RT (min)	Relative content (%)	Concentration^a^ (mg mL^−1^)	
				F-Fr.5-1	WV
1	2-Methoxy-4-methylphenol	18.35	0.96	0.08 ± 0.00	0.23 ± 0.00
2	Catechol	18.71	33.28	1.89 ± 0.06	1.08 ± 0.05
3	3-Methylcatechol	21.31	5.65	0.31 ± 0.01	0.04 ± 0.00
4	4-Methylcatechol	22.66	17.14	0.95 ± 0.03	0.12 ± 0.00
5	4-Ethylcatecol	26.45	8.32	0.44 ± 0.01	0.03 ± 0.00
6	2,3-Dimethylhydroquinone	27.41	2.71	—b	—
7	Benzoic acid, 3-hydroxy, methyl ester	28.26	1.17	—	—
8	1,2,4-Trimethoxybenzene	28.82	2.55	ND^c^	ND
9	1,3-Benzenediol, 4-propyl-	29.61	2.47	—	—
10	Benzene, 1,2,3-trimethoxy-5-methyl	30.66	2.17	—	—
	Others	—	23.58	—	—
	Total	—	100	6.1	—

a Concentration was expressed as the mean ± SD (*n* = 3).

b Not detected.

c Concentration was lower than the quantification limit (2.9 nmol mL^−1^).

**Table 2:** Catechol and catechol derivatives in F-Fr.5-2 and WV

No	Compounds	RT (min)	Relative content (%)	Concentration^a^ (mg mL^−1^)	
				F-Fr.5-2	WV
11	3-Hydroxytetrahydro-2-*H*-pyran-2-one	14.13	4.16	—b	—
12	3-Ethyl-2-hydroxy-2-cyclopenten-1-one	15.14	3.33	—	—
2	Catechol	18.59	3.62	0.20 ± 0.00	1.08 ± 0.05
13	3-Methoxycatechol	21.15	2.37	0.12 ± 0.00	ND^c^
14	2,6-Dimethoxyphenol	25.16	19.28^d^	1.68 ± 0.02	0.08 ± 0.00
15	2-Methyl-1,4-benzenediol	25.16	19.28^d^	0.42 ± 0.03	ND
16	Ethanone, 1-(3-hydroxyphenyl-)	27.20	4.24	—	—
17	1,2,4-Trimethoxybenzene	28.84	3.87	ND	ND
18	Benzoic acid, 4-hydroxy-3-methoxy-, methyl ester	30.59	4.28	—	—
19	1,2-Dimethoxy-4-*n*-propylbenzene	31.79	6.75	—	—
	Others	—	48.1	—	—
	Total	—	100	5.0	—

a Concentrations were expressed as the mean ± SD (*n* = 3).

b Not detected.

c Concentrations were lower than the quantification limit (3.5 nmol mL^−1^).

d Total relative contents of 2,6-dimethoxyphenol and 2-methyl-1,4-benzenediol.

### Dose–dependent antiviral activities of catechol derivatives

**Fig. 3:**
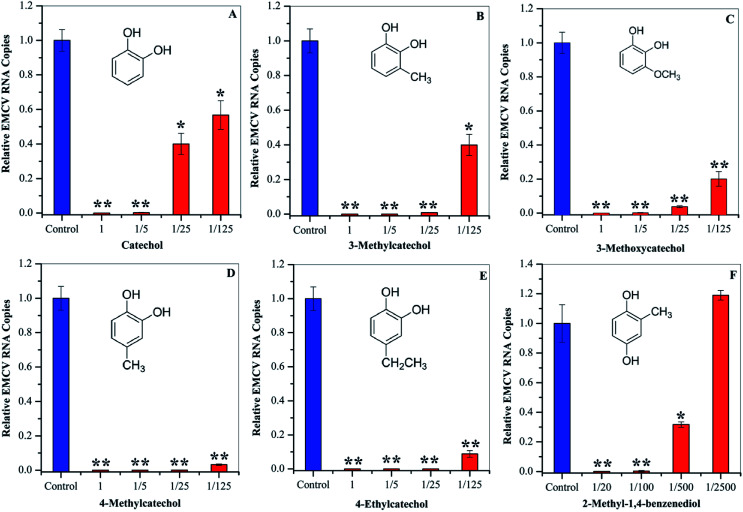
Antiviral activities of catechol (A), 3-methylcatechol (B), 3-methoxycatechol (C) 4-methylcatechol (D), and 4-ethylcatechol (E) at 1 (50 mg mL^−1^), 1/5 (10 mg mL^−1^), 1/25 (2.0 mg mL^−1^), and 1/125 (0.4 mg mL^−1^) dilutions. 2-Methyl-1,4-benzenediol (F) was assayed at 1/20 (2.5 mg mL^−1^), 1/100 (0.5 mg mL^−1^), 1/500 (0.1 mg mL^−1^), and 1/2500 (0.02 mg mL^−1^) dilutions. *P < 0.05, **P < 0.01 indicate a significant difference compared to the solvent control by student's *t*-test.

To evaluate whether the identified compounds could exert an antiviral effect on EMCV, catechol and its derivatives were assayed by incubating these compounds with the virus respectively, followed by cell infection. As shown in Fig. 3, all of these compounds efficiently decreased the virus infectivity. To evaluate the viral inhibition effects at different concentrations, catechol and its derivatives were diluted at 5-, 25-, and 125-fold from the stock solutions (50 mg mL^−1^). Dilution for 2-methyl-1,4-benzenediol was 20-, 100-, 500-, and 2500-fold since high cytotoxicity was observed at high concentrations. Catechol and 3-methylcatechol showed similar viral inactivation effects, while 4-methylcatechol, 4-ethylcatechol, and 2-methyl-1,4-benzenediol completely inactivated EMCV at concentrations of 2.5 mg mL^−1^ (20.1 μmol mL^−1^ for 4-methylcatechol). As shown in Fig. 3, all the catechol derivatives were observed to inhibit EMCV in a dose–dependent manner. At 0.4 mg mL^−1^ (3.5 μmol mL^−1^ for catechol), catechol, 3-methylcatechol, 4-methylcatechol, 4-ethylcatechol, and 3-methoxycatechol inhibited EMCV by 43.2%, 60.1%, 96.8%, 91.2%, and 79.9%, respectively, and 2-methyl-1,4-benzenediol at 0.5 mg mL^−1^ (4.0 μmol mL^−1^) inhibited EMCV by 99.7% (Table 3). The half maximal concentrations for EMCV inhibition (IC_50_) and for cytotoxicity (CC_50_) of catechols were also evaluated and are shown in Table 3. Of all the catechol derivatives, 2-methyl-1,4-benzenediol showed the strongest viral inactivation effect; however, this compound also exhibited highest cytotoxicity. From Fig. 3 and Table 3, it can be concluded that the antiviral activity of catechol derivatives is related to the substituent group on the catechol skeleton. Methyl- and ethyl- substitution in the *para* position relative to a hydroxyl group significantly increased antiviral activities. The position of substituent group also affects the antiviral activity, *e.g.*, 4-methylcatechol exerted higher activity than 3-methylcatechol (Table 3). In addition, the relative position of the two hydroxyl groups on the benzene ring is also important for the antiviral activity; 2-methyl-1,4-benzenediol showed higher viral inhibition effects than 1,2-benzenediol (catechol). However, 2-methyl-1,4-benzenediol also showed higher cell cytotoxicity than the others.

**Table 3:** Evaluations of IC_50_ and CC_50_ of catechols derivatives

Compound	IC_50_^a^ (mg mL^−1^)	CC_50_ (mg mL^−1^)	Inhibition^b^ (%)
Catechol^c^	0.67 ± 0.18	>1.03	43.2
3-Methylcatechol^c^	<0.41	0.33 ± 0.01	60.1
4-Methylcatechol	<0.41	0.28 ± 0.06	96.8
4-Ethylcatechol	<0.42	0.34 ± 0.01	91.2
3-Methoxycatechol^c^	<0.41	0.37 ± 0.09	79.9
1,4-Benzenediol, 2-methyl-	0.20 ± 0.14	<0.03	99.7

a IC_50_ is the concentration of catechols, which were incubated with the virus.

b Inhibition effects at 0.4 mg mL^−1^; the inhibition effect of 1,4-benzenediol, 2-methyl- was calculated at 0.5 mg mL^−1^.

c IC_50_ and CC_50_ of these compounds were reported in our previous research.^25^

### Inactivation of viral particles by catechol derivatives

**Fig. 4:**
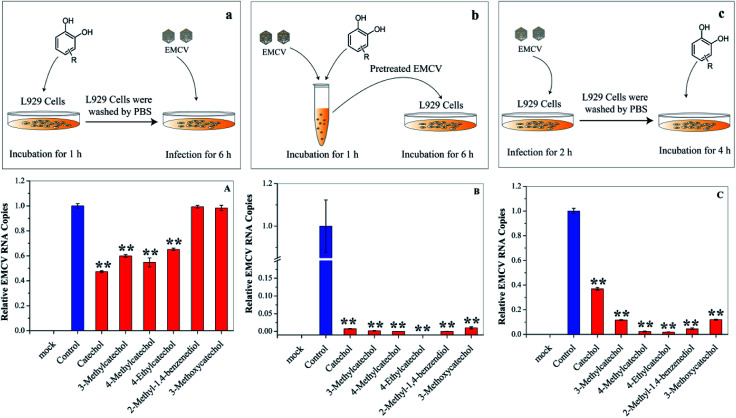
Effects of catechol derivatives on the host cell and virus. (a) and (A) L929 cells were incubated with catechol derivatives for 1 h and then washed with PBS. The pretreated cell was infected with EMCV for 6 h. (b) and (B) EMCV was first incubated with catechol derivatives for 1 h. Then, L929 cells were infected with the pretreated EMCV for 6 h. (c) and (C) L929 cells were infected with EMCV for 2 h, and catechol derivatives were then added into the culture medium. Virus RNA was quantified by qPCR. *P < 0.05, **P < 0.01 indicate a significant difference compared to the solvent control by student's *t*-test.

Each stage of virus infection may be a possible target for inhibition. To determine the stage at which EMCV was inhibited catechol derivatives were added at different times: (a) pre-incubation with cells for 1 h before virus infection, (b) mixing with the virus for 1 h, and (c) after virus adsorption. Cells pre-treated with catechol derivatives exhibited considerable protection effects against EMCV infection (Fig. 4A). The protection capacities were dependent on the structures of the compounds; catechol, 3-methylcatechol, 4-methylcatechol, and 4-ethylcatechol exhibited stronger inhibitory effects than 2-methyl-1,4-benzenediol and 3-methoxycatechol. Moreover, strong antiviral activities were observed when EMCV was pre-exposed to catechol derivatives before cell infection (Fig. 4B). Interestingly, catechol derivatives still showed significant antiviral activity two hours after viral infection (Fig. 4C). These results implied that catechol derivatives not only directly inactivated EMCV virions but also had host immune activation effect.

### Activation of the host immune system by catechol derivatives

**Fig. 5:**
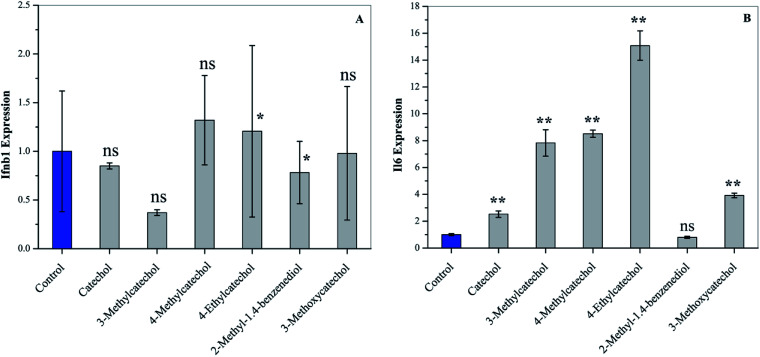
Activation of cytokines of L929 cells after treatment with catechol derivatives (25 μg mL^−1^) for 3 h. The expression levels of (A) Ifnb1 and (B) Il6 were analyzed by qPCR. *P < 0.05, ** *P* < 0.01 compared to the solvent control by student's *t*-test, ns means not significant.

Interferons (IFN) can modulate the host immune response to viral infection and inhibit viral replication.^26,27^ Transcription factors of the interferon regulatory factor (IRF) family mediate the entire type I IFN (IFN-α and -β) system, thereby supplying a primary basis for host resistance against pathogens. Among the IRF family, IRF-3 and IRF-7 stimulate the expression of type I IFN (IFN-α and -β).^28,29^ In order to confirm whether catechol derivatives can activate the host immune response, L929 cells were incubated with catechol derivatives for 3 h, and the expression level of Ifnb1 was quantified by qPCR. Fig. 5A clearly shows that Ifnb1 was not induced by any catechol treatment, which reveals that catechol derivatives did not activate IRF-3 and IRF-7. Another important transcription factor is NF-κB, which plays a critical role in antiviral defense.^30,31^ Interleukin-6 (IL6), a cytokine, was expressed upon activation of NF-κB.^32^Fig. 5B demonstrates that the expression level of Il6 was significantly increased when L929 cells were incubated with catechol derivatives, especially 3-methyl-, 4-methyl-, and 4-ethylcatechol. Pretreatment with 2-methyl-1,4-benzenediol did not increase the expression of Il6 to any degree. Thus, we can conclude that the antiviral activities of catechol and its derivatives may be ascribed to the activation of NF-κB.

### ROS quantification

**Fig. 6:**
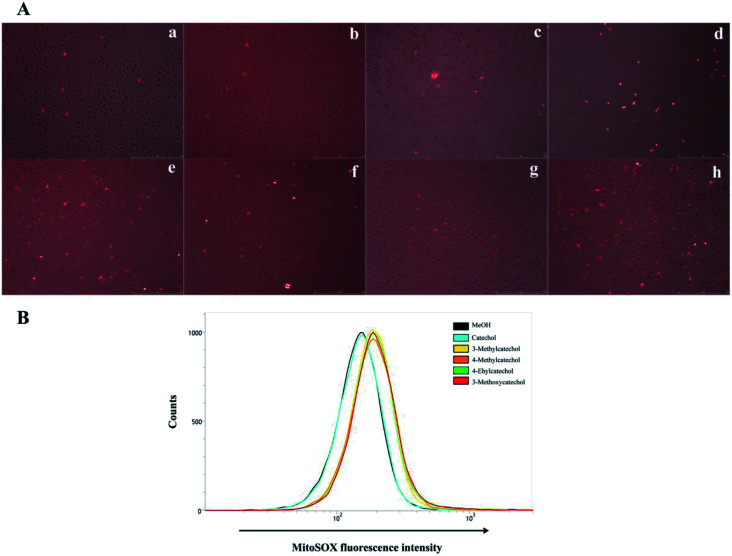
(A) Confocal fluorescence images of ROS production in L929 cells obtained by MitoSOX™. (a) Mock, (b) solvent control (MeOH), (c) catechol, (d) 3-methylcatechol, (e) 4-methylcatechol, (f) 4-ethylcatechol, (g) 2-methyl-1,4-benzenediol, and (h) 3-methoxycatechol. L929 cells were incubated with catechol derivatives for 3 h and treated with a MitoSOX™ reagent for 10 min. ROS were detected by live cell imaging (B) quantification of ROS generated from auto-oxidation of catechol derivatives. The MitoSOX™ fluorescence intensity was measured at 580 nm in L929 cells by flow cytometry.

It is reported that ROS can activate NF-κB.^33,34^ Auto-oxidation of catechol derivatives leads to the production of ROS. We also confirmed the generation of ROS by treatment of L929 cells with catechol derivatives using MitoSOX™ as an indicator. In the presence of ROS, MitoSOX™ showed red fluorescence. From the microscope images (Fig. 6A), we can clearly observe the production of ROS when cells were treated with 3-methyl-, 4-methyl-, and 4-ethylcatechol. We also quantified the amounts of ROS by determination of the fluorescence intensity of MitoSOX at 580 nm. As shown in Fig. 6B, catechol did not increase the production of ROS when compared with a solvent control, whereas 3-methyl-, 4-methyl-, and 4-ethylcatechol significantly enhanced the generation of ROS, which was consistent with the expression level of Il6. Considering these results together, we can conclude that ROS generated from the auto-oxidation of catechol derivatives resulted in the activation of NF-κB, which is important in antiviral defense. Additionally, ROS have been reported to directly disrupt viruses.^35–37^ Catechol derivatives showed viral inhibitory activities when added two hours after infection. One possible reason may be that ROS generated from auto-oxidation of catechol derivatives inactivated the newly propagated virions.

ROS have historically been considered toxic but unavoidable species during aerobic metabolism. Excessive production of ROS will lead to DNA mutations, lipid per-oxidation, and protein oxidation. Therefore, it is important to eliminate these oxidation species. However, just like a coin has two sides, ROS have also been reported to play a role in many cellular functions, such as innate immunity, signal transduction, and modification of the extracellular matrix.^38,39^ ROS produced by phagocytes play an important role in host defense against pathogens.^40^ In addition, ROS produced by NADPH oxidase regulate plant cell expansion through the activation of Ca^2+^ channels.^41^ The role of ROS in the viral life cycle is complicated and dependent on the cell and the type of virus.^37,42–44^ In our research, we find ROS can directly inactivate EMCV and also activate NF-κB, which mediates the expression of Il6. However, in some cases, ROS may facilitate or even enhance the replication of viruses. Elucidation of the roles of ROS in antiviral activity of catechol and its derivatives awaits further study.

### Chemical index for production of antiviral compounds

Due to the lack of scientific basis, there are no concrete strategies for conversion woody biomass into valuable antiviral compounds. In this study we showed that catechol and its derivatives are the major antiviral compounds in WV, and position and structure of substituent group attached to the aromatic ring significantly affect viral inactivation activity. Thus, this research provides the important chemical indices for optimizing production of antiviral compounds. For instance, pyrolysis conditions producing higher amounts of potent antiviral catechol derivatives are expected to exhibit stronger total antiviral activities. New chemical basis was obtained for conversion of woody biomass into antiviral agents.

## Conclusion

The pyrolysis product, WV derived from Japanese larch showed strong antiviral activity against EMCV. Catechol and its derivatives with different substituent group were major antiviral compounds in the WV. These components exhibited different levels of antiviral activity depending on the position and structure of the function groups attached to an aromatic skeleton. It was found that the catechol derivatives generated ROS, inactivated EMCV directly and induced production of cytokine Il6. These findings will lead to the synthesis of catechol-based antiviral agents with high activity and development of the process for producing antiviral compounds from lignocellulosic biomass.

## Conflicts of interest

There are no conflicts to declare.

## Supplementary Material
